# Gingivitis and periodontitis among Indian patients diagnosed using trefoil factor 3

**DOI:** 10.6026/9732063002001065

**Published:** 2024-09-30

**Authors:** Sai Priyanka K., Veerendranath Reddy P., Rajashree D., Arshad Jamal Sayed

**Affiliations:** 1 Department of Periodontics, Private Practitioner, Hyderabad, Telangana, India; 2 Department of Periodontics, Panineeya Institute of Dental Sciences and Research Centre, Hyderabad, Telangana, India; 3 Department of Peridontology and Implant Dentistry, College of Dentistry, Qassim University, Saudi Arabia

**Keywords:** Diagnostic marker, gingivitis, periodontitis, trefoil factor 3

## Abstract

Recently there has been an increased interest on the identifying and preventing the disease with profuse biomarkers. There are very few studies done in understanding the relationship of TFF3 levels in the diseased condition. Therefore, it is of interest to determine effect of non-surgical periodontal therapy on trefoil factor levels in patients with gingivitis and periodontitis. Study sample consisting of 40 subjects of which 20 with gingivitis and 20 with moderate chronic periodontitis were included. The clinical parameters assessed were gingival index, Plaque index, and Probing Depth and Clinical attachment levels. Enzyme linked immunosorbent assay was used to evaluate Serum Trefoil factor 3 (TFF3). Intra group comparisons in gingivitis and periodontitis group have shown that all clinical and biochemical parameters have significantly reduced from base line to 3 months. Increased serum TFF3 concentrations were observed after non-surgical periodontal therapy in both gingivitis and chronic periodontitis group (p<0.001). In conclusion clinical and biochemical analysis of serum TFF3 revealed its influence on periodontal disease activity. Elevated serum Trefoil factor 3 levels showed a strong association with decrease in clinical parameters taken after the therapy.

## Background:

The well-known inflammatory disease of the supporting tissues of the teeth which is chronic in nature is Periodontitis, commencing with copious factors [1]. Recently there has been an increased interest on the identifying and preventing the disease with profuse biomarkers. The Pancreatic spasmolytic peptide was the term coined for soluble proteins so called Trefoil factors (TFFs) whose initial member were analyzed thirty years back in 1980's [2]. The three trefoil factor peptides are TFF1, TFF2 and TFF3 from which the mucous epithelium secretes the three mammalian trefoil factors with mucus gel [3]. The small secretory products from various mucin producing cells of mucous membrane in the body have a protective role in function. The TFF domain consists of three intra-chain disulfide bonds where TFF1 and TFF3 are either homodimers or heterodimers and they have an individual cystein residue at the N-terminal end of the peptide chain [4]. Numerous cells synthesize the trefoil factor peptides such as gastric mucosal lining secrets TFF1 whereas the Brunner's gland of duodenum synthesizes TFF2 and intestinal goblet cells synthesize TFF3 [5]. The only determined peptide in whole saliva is TFF3 which is expressed by mucin MUC7 by the serous cells of the submandibular gland [6]. Till date there are very few studies done in understanding the relationship of TFF3 levels in the diseased condition. Therefore, it is of interest to measure serum trefoil factor 3 at baseline and 3 months after following nonsurgical periodontal therapy in patients with Gingivitis and Chronic Periodontitis.

## Materials and Methods:

This prospective study was conducted between December 2015 to December 2017 involving 40 patients with 20 gingivitis and 20 periodontitis patients to evaluate the serum trefoil factor level 3 before and after three months of Nonsurgical Periodontal Therapy. The study was approved from institutional ethics committee and consent from all the participants.

## Inclusion & Exclusion criteria:

Patients of age between 20 to 50 years with a Gingival Index - 1.1 to 2 and Bleeding on Probing at ≥ 20 % sites and for periodontitis patients with probing Depth of > 4 mm and clinical attachment loss of > 2 mm along with presence of at least 20 permanent teeth were included in the study protocol. The Exclusion criteria include 1) those with presence of gingival recession and clinical attachment loss 2) those with presence of furcation 3) those with systemic infections like diabetes and Smokers 4) those who received antibiotic therapy within the previous 3 months 5) those who underwent periodontal therapy within 3 months 6) those who were pregnant and lactating mothers.

## Data collection and clinical parameters:

Forty subjects were randomly allocated into two groups Group A and Group B. Group A comprised of the 20 subjects with gingivitis and Group B comprised of 20 subjects with chronic periodontitis. Data was collected at baseline (before therapy) and 3 months after therapy. At baseline and after 3 months the clinical parameters which were evaluated were gingival index, Modified Plaque index [7]. Probing depth and Clinical attachment level were assessed [8].

**Figure 1:**
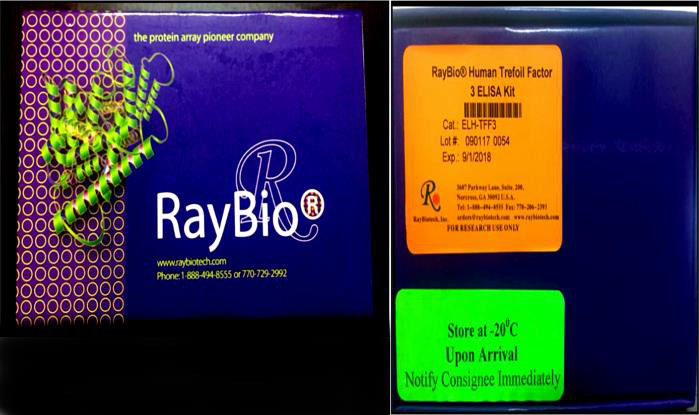
Ray Bio Human TFF-3 ELISA kit

**Figure 2:**
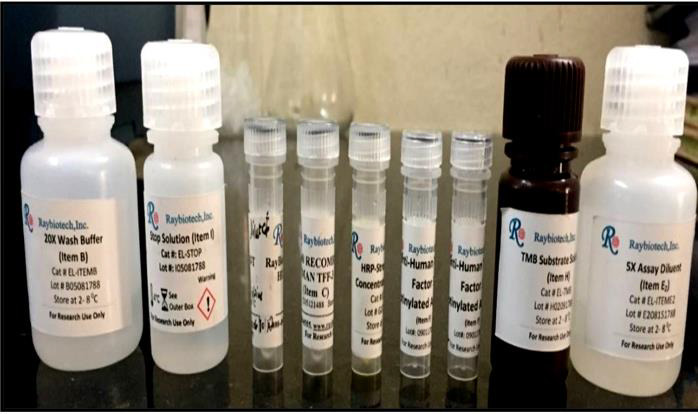
Reagents used in ELISA

After all the clinical parameters were evaluated 2ml of blood was collected in test tube and the test tube containing 2ml blood was subjected to centrifugation at 3000rpm for 10 min. The supernatant straw colored fluid (serum) was separated into storage vials (Eppendorf tubes) for serum Trefoil Factor 3. Group A comprised of the 20 subjects with gingivitis who underwent scaling and root planning and Group B comprised of 20 subjects with chronic periodontitis who underwent scaling and root planning. All the patients were given Oral hygiene instructions. The entire procedure from determining the clinical parameters to collection of serum was repeated after 3 months from the day of scaling and root planing. A TREFOIL FACTOR 3 assessment was done by commercial kit by Ray Bio Human TFF-3 ELISA kit (Figure 1) for all the collected samples. Reagents used in ELISA are shown in Figure 2.

## Statistical analysis:

Data was analyzed using SPSS version 22.0. Data was summarized by Mean ± SD for continuous data and Median ± IQR (Inter-Quartile Range) for score data. Data was summarized by Percentages for categorical data. The comparison between base line and 3 months was done by paired t-test for continuous data and Wilcoxon signed Rank test. The comparison between two groups was done by unpaired t-test for continuous data. The association between two groups was done by chi-square test/Fishers exact test for categorical data. All p-values less than 0.05 were considered as statistically significant.

## Results:

Table 1 describes that there was a statistically significant reduction in mean values of all the clinical and biochemical parameters from baseline to 3-months within gingivitis group. The mean Gingival Index (GI) values have reduced from 3.0 at baseline to 1.60 at 3 months. The mean Plaque index (PI) values have reduced from 2.65 at baseline to 1.60 at 3 months. The mean trefoil factor 3 values has increased from 0.63 at baseline to 2.73 at 3-months after treatment with p-value<0.001 which was statistically significant. Table 2 describes there was a statistically significant reduction in mean values of all the clinical and biochemical parameters from baseline to 3-months within Periodontitis group. The mean Gingival Index (GI) values have reduced from 1.60 at baseline to 1.50 at 3 months after treatment with a p-value<0.001. The mean Plaque index (PI) values have reduced from 2.50 at baseline to 1.50 at 3 months after treatment with p-value <0.001. The mean Probing pocket depth (PPD) values have reduced from 4.85 at baseline to 2.75 at 3 months after treatment with p-value <0.001 which was statistically significant. The mean clinical attachment (CAL) values have reduced from 4.45 at baseline to 1.85at 3-months after treatment with p-value<0.001 which was statistically significant. The mean trefoil factor 3 values has increased from 0.61 at baseline to 2.55 at 3-months after treatment with p-value<0.001 which was statistically significant. Table 3 describes that there is no statistically significant difference between the PI and GI at 3 months in both the groups. There is no statistically significant difference between the TFF 3 levels at 3 months in both groups with higher values in group A.

## Discussion:

The increase in vascular flow, permeability and influx of cells from peripheral blood to the gingival crevice leads to inflammation of gingiva called as Gingivitis [9]. The cytokines and immuno-globulins appear at the lesion as an antigen specific response. The tissue destruction initially is restricted to epithelial cells and collagen fibres from the connective tissue. Later on the inflammatory process may reach tissue leading to Periodontitis [10]. Several studies have demonstrated that non-surgical periodontal therapy (*e.g.*, scaling and root planing) produces enhance improvement in periodontal health as measured by reductions in probing depth (PD) and bleeding on probing (BOP) and gain in clinical attachment level (CAL) [11] where the treatment success is gauged by improvements in clinical variables and reductions in subgingival microbial counts [12]. The ultimate goal of periodontal diagnostic procedure is to provide sufficient knowledge to the clinician to evaluate the present periodontal disease type and severity in order to serve for the treatment planning and periodontal maintenance. The traditional diagnostic procedures such as probing depth, Clinical attachment levels, Plaque index and Alveolar bone assessment by radiographs evaluate only disease history but the current status cannot be assessed. Advances in diagnostic research are moving towards methods where periodontal risk can be identified and quantified by objective measures such as Biomarkers.

Thim in 1989 named a new group of growth factors like peptides as "Trefoil". The TFF encompasses a group of low molecular weight, soluble proteins that share a common feature - a three-looped trefoil-like structure formed through inter-chain disulphide bonding which is the basis for the extraordinary resistance of these peptides to hydrolysis and proteolysis [13]. TFFs perform various functions, which include proliferation, anti-apoptosis, wound repair, regeneration, neo-vascularization and mucin interaction. In our study 40 patients with gingivitis and periodontitis with moderate disease activity were enrolled and evaluated for serum TFF3 after Non-surgical periodontal therapy. Quantitative measurements of Trefoil Factor 3 have been important tools for elucidating the biological functions of periodontium and this strategy has been utilized to explore their role as Biomarkers. Clinical studies on utility of trefoil factor as a marker of disease activity in periodontitis patients are sparse.

According to our observations, there are two important findings in the present study. First, serum TFF3 concentration levels were negatively co-related to clinical parameters such as gingival index, Plaque index, Probing depth and Clinical attachment loss. There was increase in TFF3 levels after therapy in both the groups. It remains unclear how bacterial count can be relate to reduced TFF3 levels. The most likely explanation is that the bacterial infection induces chronic inflammatory response that in turn inhibits TFF3 gene expression. Secondly, increased TFF3 levels has been observed in both gingivitis and periodontitis group post operatively with nonsurgical periodontal therapy with higher values in group A *i.e.* gingivitis group with a mean of 2.73 in Group A and 2.55 in Group B. Intragroup comparison in the present study shows that there was a statistically significant reduction in mean values of all the clinical parameters from baseline to 3-months in both groups *i.e.* GROUP A and GROUP B. The mean Gingival Index have reduced from 3.00 at baseline to 1.60 at 3 months with p value <0.001 which was statistically significant in Group A and 1.60 at baseline to 1.50 at 3 months with p value <0.001 statistically significant in Group B. The mean Plaque Index have reduced from 2.65 at baseline to 1.60 at 3 months with p value <0.001 which is statistically significant in Group A and 2.50 at baseline to 1.50 at 3 months which is statistically significant in Group B indicating significant improvement in reduction of gingival and plaque index due to non-surgical periodontal therapy.

In periodontitis group i.e. Group B, the mean Probing depth (PD) have reduced from 4.85 at baseline to 2.75 at 3 months with a p value <0.001 which is statistically significant. The mean Clinical attachment (CAL) values have reduced from 4.45 at baseline to 1.85 at 3 months which is statistically significant (<0.001), which is in accordance with Badersten *et al.* who also observed a considerable pocket reduction following nonsurgical periodontal therapy involving root instrumentation with hand or ultrasonic instruments under local anesthesia [14]. The greatest change in probing depth reduction and gain in clinical attachment occurs within 1-3months post-scaling and root planing, although healing and maturation of the periodontium may occur over the following 9-12months [15]. Thus, evaluation of the response of the periodontium to scaling and root planing should be performed no earlier than 4weeks following treatment. The function of salivary trefoil factor 3 (TFF3) in patients with gingivitis and periodontitis was identified by Meesala *et al.* They came to the conclusion that patients with moderate-to-severe periodontitis may benefit from using the estimation of TFF3 levels to help with treatment approach decisions [16]. There is a negative connection between salivary TFF3 levels and inflammatory mediators. TFF3 is an important biomarker to determine the activity and association of periodontal and systemic diseases [17]. Established ELISA will be a valuable tool for facilitating the investigation of the physiological roles and the diagnostic values of TFF3 in oral diseases [18]. Hormdee *et al.* demonstrated the effects of periodontal disease on the production of salivary TFF3 peptides. Interestingly, nonsurgical periodontal treatment also affected the recovery of salivary TFF3 peptides [19]. Further the treatment aimed in arresting the periodontal destruction resulted in statistically significant increase in the levels of TFF3 in serum of both the groups. TFF3 levels in the serum of gingivitis and periodontitis patients differed from pre operatively to post operatively suggesting that TFF3 is a novel biomarker easy to be measured in the clinic. Further longitudinal studies in larger population are required to quantitatively assess the relationship between trefoil factor and severity of periodontitis. Although the detailed mechanism of action of the trefoil peptides is just beginning to be uncovered, one might start to consider possible ways of interfering in epithelial restitution processes when things go wrong.

## Conclusion:

Trefoil factor is a well-known serum marker which proved to be decreased in many disease conditions including periodontitis. The above results has proved that Trefoil factor 3 expression has decreased at baseline and increased after non-surgical periodontal therapy which confirmed that Trefoil factor 3 could be a unique biomarker in interpreting the disease levels.

## Figures and Tables

**Table 1:** Intra group comparison of clinical and biochemical parameters in group A

**Parameter**	**Groups**	**N**	**Minimum**	**Maximum**	**Mean**	**Std. Deviation**	**p value**
GI	Group A Baseline	20	3	3	3	0	< 0.001**
	Group A 3 months	20	1	2	1.6	0.5	
PI	Group A Baseline	20	2	3	2.65	0.49	< 0.001**
	Group A 3 months	20	1	2	1.6	0.5	
TFF 3	Group A Baseline	20	0	2.53	0.63	0.85	< 0.001**
	Group A 3 months	20	0.93	9.45	2.73	1.94	
**-Highly significant (p<0.001)

**Table 2:** Intra group comparison of clinical and biochemical parameters in group B

**Parameter**	**Groups**	**N**	**Minimum**	**Maximum**	**Mean**	**Std. Deviation**	**p value**
GI	Group B Baseline	20	1	2	1.6	0.5	< 0.001**
	Group B 3 months	20	1	2	1.5	0.513	
PI	Group B Baseline	20	2	3	2.5	0.513	<0.001**
	Group B 3 months	20	1	2	1.5	0.513	
Probing Depth	Group B Baseline	20	3	7	4.85	1.19	<0.001**
	Group B 3 months	20	2	5	2.75	0.88	
CAL	Group B Baseline	20	3	8	4.45	0.5	<0.001**
	Group B 3 months	20	1	3	1.85	0.5	
TFF 3	Group B Baseline	20	0.284	2.743	0.61	0.659405	<0.001**
	Group B 3 months	20	40.31	58.44	2.55	5.71328	
*-Highly significant (p<0.001)

**Table 3:** Inter group comparison of clinical and biochemical parameters in group A and group B

**Parameter**	**Groups**	**N**	**Minimum**	**Maximum**	**Mean**	**Std. Deviation**	**p value**
GI	Group A 3 months	20	1	2	1.6	0.5	0.530 NS
	Group B 3 months	20	1	2	1.5	0.513	
PI	Group A 3 months	20	1	2	1.6	0.5	0.530 NS
	Group B 3 months	20	1	2	1.5	0.513	
Probing Depth	Group A 3 months	20	-	-	-	-	-
	Group B 3 months	20	2	5	2.75	0.88	
CAL	Group A 3 months	20	-	-	-	-	-
	Group B 3 months	20	1	3	4.45	0.5	
TFF 3	Group A 3 months	20	80.91	111.1	2.73	7.97	< 0.001**
	Group B 3 months	20	40.31	58.44	2.55	5.71328	
Not recorded, NS - not significant (p>0.05),
**-Highly significant (p<0.01)
